# Interactions between Multiple Organs and Nutritional Metabolism in the Development and Progression of Metabolic Dysfunction-Associated Steatotic Liver Disease

**DOI:** 10.3390/nu15234910

**Published:** 2023-11-24

**Authors:** Masato Yoneda, Atsushi Nakajima

**Affiliations:** Department of Gastroenterology and Hepatology, Graduate School of Medicine, Yokohama City University, Yokohama 236-0004, Japan

## 1. Introduction

Steatotic liver disease (SLD) is a condition characterized by an accumulation of fat in the liver. Metabolic dysfunction-associated steatotic liver disease (MASLD) is a type of SLD diagnosed when at least one abnormality is present among cardiometabolic risk factors, such as BMI, waist circumference, blood glucose or HbA1c levels, blood pressure, triglycerides, and HDL cholesterol [1]. It is also diagnosed when alcohol consumption is <140 g and <210 g per week for women and men, respectively. This condition was previously known as non-alcoholic fatty liver disease (NAFLD) [1,2]. Initially, in 1980, Ludwig et al. introduced the concept of non-alcoholic steatohepatitis (NASH) as a “liver disease that histologically resembles alcoholic hepatitis and may progress to cirrhosis” [3]. Subsequently, the term “non-alcoholic fatty liver disease (NAFLD)” was coined by Schaffner et al. in 1986 to describe fatty liver diseases not caused by alcohol consumption [2]. However, due to the stigmatizing nature of the terms “alcoholic” and “fatty”, the European Association for the Study of the Liver (EASL), the American Association for the Study of Liver Diseases (AASLD), and the Latin American Association for the Study of Liver Diseases (ALEH) jointly issued a consensus statement in June 2023 to rename NAFLD as MASLD and NASH as metabolic dysfunction-associated steatohepatitis (MASH) [1,2,3].

MASLD has garnered attention as a systemic disease due to its pathogenic mechanisms, adverse outcomes, and interactions with other organs [4]. If MASLD and their associated complications are not proactively screened for, they may progress unnoticed. However, due to a large number of potential patients, gastroenterologists and hepatologists are unable to manage these patients alone. Therefore, collaborative efforts involving specialists from various fields, including family doctors, nutritionists, and pharmacists, are necessary for the treatment of MASLD.

This Topic “Multiple Organ Cross-Talk and Nutrition Metabolism in the Development and Progression of NAFLD/MAFLD” in *Nutrients* focuses on the complex interplay of MASLD with multiple organs, emphasizing its various organ associations. It aims to explore the diverse organ-related aspects of MASLD and promote its clinical management through collaboration among multiple healthcare professionals.

The most crucial factor in considering MASLD as a systemic disease is its strong association with obesity, type 2 diabetes, and/or insulin resistance. MASLD and obesity, as well as type 2 diabetes, can both act as causative factors and potentially influence each other, and are most commonly linked to the development and pathological progression of MASLD. In 2019, the American Diabetes Association (ADA) recommended that diabetic patients with fatty liver or abnormal liver function should undergo evaluation for NASH and fibrosis [5]. The 2023 ADA consensus statement includes additional details concerning the diagnosis and risk stratification of MASH in primary care and diabetes clinics, such as the use of the fibrosis-4 (FIB-4) index to assess the risk of liver fibrosis. It also explains the rationale behind fibrosis risk stratification in diabetic patients and provides recommendations for referring patients to a gastroenterologist or hepatologist [6]. Furthermore, pioglitazone, sodium–glucose co-transporter 2 (SGLT-2) inhibitors, and glucagon-like peptide (GLP)-1 receptor agonists have been reported to improve both diabetes and MASLD. Since many patients with type 2 diabetes also have MASLD, it is crucial to understand how to identify patients who require collaboration between diabetologists, gastroenterologists, and hepatologists.

Notably, cardiovascular disease (CVD) can have a profound impact on the prognosis of MASLD. Concurrently, MASLD has been significantly associated with an increased cardiovascular risk, with an odds ratio of 1.84, even after adjusting for age, sex, smoking history, diabetes history, hemoglobin A1c (HbA1c), low-density lipoprotein (LDL) cholesterol, liver enzymes, and medications [7]. Several studies have suggested that hepatic fibrosis plays a role in atherogenesis. Patients with fibrotic MASH may develop CVD and exhibit accelerated atherosclerosis, possibly due to increased hepatic production of prothrombogenic factors, such as vascular endothelial growth factor, hypoxia-inducible factor, intracellular adhesion molecule-1, vascular adhesion molecule-1, and fetuin-A [8].In addition to atherosclerosis, MASLD can also impact cardiac function. The association between MASLD and CVD is not limited to ischemic heart disease but also extends to heart failure and primarily diastolic failure, which is becoming increasingly prevalent among older patients. Therefore, the prevention of heart failure and atherosclerotic disease, both in primary and secondary care, requires collaboration between gastroenterologists, hepatologists, and cardiologists.

The potential systemic diseases associated with MASLD not only include obesity, diabetes, and heart disease but also various other conditions such as kidney, muscle, and mental health issues. While providing a brief overview, this Editorial will mention some research findings related to the kidneys, mental health, muscles, and dental health. Several longitudinal studies have reported that MASLD and fibrosis development in MASLD are risk factors for new-onset CKD [9,10]. A systematic review of ten articles involving 2,041,752 patients with MASLD reported that depression was significantly associated with MASLD, and comorbidity was associated with increased annual mortality [11]. In addition, the prevalence of MASLD is increased in patients with sarcopenia, and the presence of sarcopenia increases the risk of developing MASH and liver fibrosis [12]. Multifaceted intervention coordinated by a team of collaborating physicians, exercise therapists, dietitians, and nurses can facilitate a sustained lifestyle improvement in patients with MASLD.

To summarize, MASLD is rapidly emerging as the leading cause of hepatocellular carcinoma and is also linked to an increased risk of CVD, making it a significant health concern from both medical and socioeconomic perspectives. MASLD is a systemic disease that necessitates the involvement of numerous medical professionals. It is expected that a comprehensive understanding of the various pathologies affecting the body in the development of MASLD, as well as the adverse effects that MASLD has on the entire body, through the collaboration of multiple specialists, will lead to the development of more effective treatment strategies and contribute to the elucidation of its pathogenesis. Multidisciplinary collaboration, involving professionals with diverse specialties working together towards a common goal, enhances patient care by integrating perspectives from multiple experts and facilitating the exchange of opinions. Moreover, given the global aging population, disasters, and infectious disease outbreaks, there may be a worldwide shortage of healthcare professionals, which could be mitigated by promoting collaboration and cooperative medicine.
